# Association of Polymorphisms in RANK and RANKL Genes with Osteopenia in Arab Postmenopausal Women

**DOI:** 10.1155/2020/1285216

**Published:** 2020-12-09

**Authors:** Saba Abdi, Ihtisham Bukhari, Mohammed G. A. Ansari, Rawan A. BinBaz, Abdul Khader Mohammed, Syed Danish Hussain, Naji Aljohani, Nasser M. Al-Daghri

**Affiliations:** ^1^Biochemistry Department, College of Science, King Saud University, Riyadh 11451, Saudi Arabia; ^2^Chair for Biomarkers of Chronic Diseases, College of Science, King Saud University, Riyadh 11421, Saudi Arabia; ^3^Laboratory X, The 5th Affiliated Hospital of Zhengzhou University, Zhengzhou, Henan Province, China; ^4^Sharjah Institute for Medical Research, University of Sharjah, 27272 Sharjah, UAE; ^5^Obesity, Endocrine and Metabolic Center, King Fahad Medical City, Riyadh 59046, Saudi Arabia

## Abstract

The RANKL/RANK/OPG pathway regulates bone remodelling and turnover. However, the genetic background of bone mineral density (BMD) and osteopenia in Saudi postmenopausal women is yet to be studied. We studied the genetic polymorphism of *RANKL/RANK/OPG* with BMD and other associated factors in Saudi postmenopausal osteopenic women. A total of 439 (223 osteopenia and 216 control) postmenopausal women were recruited from the orthopaedic department of the King Khalid University Hospital, Riyadh, KSA. Genetic variants of *RANK* (rs1805034 and rs35211496), *RANKL* (rs2277438 and rs9533156), and *OPG* (rs2073618 and rs3102735) were genotyped using RT-PCR. Anthropometrics, bone mineral density, and other bone markers were measured. The levels of bone turnover markers, PTH, and RANKL were found to be significantly different between control and the osteopenia group. The odds ratio of 2.37 (1.00–5.69) for *RANK* SNP (rs1805034) indicates that subjects with CC genotype are more vulnerable to developing osteopenia as compared to subjects with TT genotype. Similarly, for *RANKL* SNP (rs2277438), the significant odds ratio of 20.56 (9.82–43.06) indicates that the subjects with GG genotype are at significantly higher risk of having osteopenia compared with the AA genotype subjects. In addition, G allele in rs2277438 also found to be a risk factor for osteopenia 4.54 (3.18–6.49) compared with A allele. However, none of the OPG genotypes shows association with osteopenia. The association of *RANK* polymorphisms with osteopenia shows its clinical importance in the diagnosis and prognosis of the bone diseases; here, we suggest that the subjects with *RANK* and *RANKL* polymorphisms may develop osteoporosis.

## 1. Introduction

Low bone mineral density (osteopenia) may cause osteoporosis but not all osteopenia patients develop osteoporosis [1, 2]. Due to the loss of estrogen, it occurs more frequently in postmenopausal women. It can also be caused due to some lifestyle-associated factors like lack of exercise, alcoholism, smoking, excessive use of glucocorticoids, and exposure to harmful radiations [3, 4]. Some other physical factors like nonweight-bearing sports such as bicycling or swimming can also trigger osteopenia. There are few chances to develop osteopenia if a person practices sports like running or cardio exercises and muscle building exercises [5–7]. Multiple studies indicated that various factors including genetic, environmental, and their mutual cross talks could cause osteoporosis and osteopenia [8, 9]. Previous genome-wide association studies identified several genetic regions that influence BMD [10]. However, these genetic variants explained a small fraction of the variations in bone density, and none was shown to have a definite influence on the risk of fracture. Physiological maxillary bone remodelling and orthodontic tooth movement (OTM) require activation of the *RANK/RANKL/OPG* system [11]. RANKL produced by osteocytes mediates the dietary calcium in bones [12]. Keeping in sight the function of the RANK/RANKL/OPG with BMD, we studied the genetic polymorphism of these genes with BMD and other associated factors in Saudi postmenopausal osteopenic women.

## 2. Materials and Methods

### 2.1. Subjects

A total of 439 (223 osteopenia and 216 control) postmenopausal women were recruited from the orthopaedic department of the affiliated hospital of King Khalid University, Riyadh, Saudi Arabia. A generalized prestructured questionnaire was filled up by every individual. Written and verbal description of the project was given to all subjects; informed written consent was obtained from individuals willing to participate in the study. A comprehensive physical examination was performed for all participants. To scrutinize genetic cause of the patients, we set the following exclusion criteria: regular use of steroids, cortisone, etc.; calcium, mineral oil and multivitamins, medication for weight loss, antacids, laxatives and diuretic medicines, patients having signs of metabolic disorder, hyper or hypoparathyroidism; and chronic renal diseases. An ethical review board of King Saud University had approved all the sampling procedures and experimental procedures. This study was performed by strictly adhering to the guidelines and regulations of the Helsinki declaration 1964 and its latest amendments.

### 2.2. Blood Sampling and Anthropometry

Blood sampling and anthropometrics were performed after 8-12 hours of complete fasting. Anthropometric data that were collected from all participants include blood pressure (BP), height and weight, and waist and hip circumference ratio (WHR). Body mass index (BMI) was calculated according to the standard equation (kg/m^2^). A total 5-10 cc blood sample was collected in tubes containing EDTA (for genomic DNA extraction) and in tubes having no anticoagulant (for serum samples).

### 2.3. Biochemical Analysis

Blood lipids and glucose levels were analyzed using a chemical analyzer (Konelab, Finland). ELISA was performed for serum 25(OH)D (IDS Ltd., Boldon Colliery, Tyne & Wear, UK) with 5.3% inter- and 4.6% intra-assay variation. The serum insulin level was measured at (Luminex xMAPW Technology platform) (Luminexcorp, Texas). Variations in inter- and intra-analysis were set <21% and 1.4–7.9%, respectively. The homeostasis model of insulin resistance (HOMA-IR) was defined as fasting insulin (IU) × fasting glucose (mmol/L)/22.5. HOMA-*β* secretion (%) was determined as 20 × fasting insulin (IU)/(fasting blood glucose − 3.5).

### 2.4. Genetic Analyses for RANK, RANKL, and OPG Polymorphisms

DNA was extracted by using DNeasy (Qiagen, Hilden, Germany) genomic DNA extraction kit, while concentration and purity were determined by using the Nanodrop spectrophotometer. Two polymorphisms from each gene were selected: *RANK* (rs1805034 and rs35211496), *RANKL* (rs2277438 and rs9533156), and *OPG* (rs2073618 and rs3102735). A TaqMan genotyping assay, PCR program, and allelic discrimination of selected polymorphisms were done by following the protocol mentioned in our previous study by using ABI TaqMan genotyping kit at real-time PCR (Applied Biosystems, Foster City, CA) [13].

### 2.5. Statistical Analysis

Data were statistically analyzed by SPSS (version 21.01, IBM, NY, USA). Mean ± standard deviation was used to express all the variables. Normality assumption was checked by the Kolmogorov-Smirnov test, and non-Gaussian variables were transformed logarithmically. *t*-test and Mann-Whitney *U* test were used for normal and nonnormal variables, respectively, to compare cases and control to see significant differences. Odds of osteopenia were obtained using logistic regression taking osteopenia as dependent while SNPs as dependent variables. A *p* < 0.05 was taken as statically significant.

## 3. Results

The mean age of the control group was significantly lower than that of the osteopenia group (53.5 ± 6.0 versus 55.8 ± 8.0; *p* = 0.001). The median menopausal age was 4.0 (2.0-7.0) in the control group and 6.0 (3.0-10.0) in the osteopenia group (*p* < 0.001). Furthermore, mean systolic BP of 130.0 ± 18.3 was elevated in the osteopenia group as compared to their healthy counterparts with 124.8 ± 17.8 (*p* = 0.004). Osteopenia and control groups had no significant difference in the values of the BMI, WHR, and diastolic BP. Among bone turnover markers, PTH and RANKL showed significant differences between control and osteopenia groups. PTH was significantly higher in osteopenia group with median 15.4 (9.6-27.5) as compared to their healthy counterparts with median 11.0 (6.6-19.1) (*p* = 0.004), whereas RANKL was significantly higher in the control group with median 34.3 (20.6-66.3) as compared to osteopenia group with median 24.0 (18.5-39.4) (*p* = 0.001). The rest of the bone turnover markers showed no significant differences between the control group and osteopenia subjects. Among interleukins, only IL6 level was found to have significantly increased in the osteopenia group with median 12.1 (6.4-25.6) as compared to 7.5 (3.2-25.5) in the control group (*p* = 0.013). TNF-*α* was also significantly higher in the osteopenia group with a median of 1.9 (1.4-2.6) as compared to the control group with a of median 1.6 (1.1-2.3) (*p* = 0.032). Furthermore, both groups have shown no significant difference in levels of glucose, lipids, and growth factors (Table 1).

Furthermore, the relationship between osteopenia and selected SNPs of RANK, RANKL, and OPG genes was studied. For the SNP rs1805034 in *RANK*, a significant odds ratio of 2.37 (1.00–5.69) was observed, which indicates that individuals carrying CC genotype were susceptible to develop osteopenia as compared to the carriers of TT genotype. The odds ratio of 2.37 (*p* = 0.05) suggests that individuals with CC genotype are more than twice as likely to have osteopenia as individuals with TT genotype (Table 2). C allele in s1805034 also shown borderline significance as a risk of osteopenia with an odds ratio of 1.51 (0.97–2.36) (*p* = 0.07).

Similarly, for rs2277438 in *RANKL*, a significant odds ratio of 20.56 (9.82–43.06) indicates that individuals with GG genotype have approximately 20.56 times chances of developing osteopenia than the subjects with AA genotype (*p* < 0.001). Further analysis showed that subjects with G allele in rs2277438 are significantly prone to have osteopenia as compared with subjects with A allele with an odds ratio of 4.54 (3.18–6.49) (*p* < 0.001) (Table 3).

CG genotype in rs2073618 shows borderline significance which suggests that it provides a protective effect against osteopenia (*p* = 0.07). Further analysis showed that G allele also provides protective effect with border significance (*p* = 0.06) and odds ratio of 0.61 (0.36-1.01) (Table 4).

## 4. Discussion

In current investigations, the *RANK* variants show a significant nominal difference in the allelic distribution of patients compared with the controls having a possible effect of the risk of osteopenia. Thus, the carriers of the homozygous genotype (CC) may present a risk to develop a bone mineral deficiency and osteopenia in menopausal women. Concerning to *RANKL* polymorphism, the possible involvement of rs2277438 in BMD and osteopenia pathogenicity was seen in our analyses. Notably, having GG genotype found to be a risk factor for osteopenia in healthy women as compared to AA genotype.

RANK, the receptor for RANKL, can transduce osteoclastogenic signals by ligating with anti-RANKL and anti-RANK antibodies which are needed for osteoclast formation. Knockout mice of the *RANK* revealed symptoms of non-TRAP+ osteoclastic osteoporosis [14]. The genetic causes of the familial expansile osteolysis and early-onset Paget's diseases were located at *RANK* loci [11, 12]. The *RANK* polymorphisms can be a genetic cause of low BMD [10, 15, 16]. It has been reported that *RANK*+34863G>A (rs12458117) and intronic +35928insdelC were significantly correlated with the BMD of the lumbar spine in Korean postmenopausal women [15]. +35966insdelC in *RANK* showed a significant association with BMD of the lumbar spine and femoral neck in postmenopausal women [16]. The loci at 18q21, nearby *RANK*, were associated with osteoporotic fractures in 5861 Icelandic individuals [10]. A recent study demonstrates that genetic polymorphisms of the RANK gene might cause BMD variance and osteoporosis in Saudi postmenopausal women [17].

Gennari et al. found exclusive associations of VDR gene SNP rs2228570 (*Fok*I polymorphism) with the BMD of hip and lumbar spine *T*-score [18]. A recent study investigated the effects of the antiosteoporosis treatment in 418 Southern Italian postmenopausal women and revealed that the carriers of *Fok*I TT genotype at the baseline had lower BMD and were more responsive to alendronate therapy, as compared to TC and CC genotype carriers of this SNP [19]. It is noteworthy that from the present analyses, a novel indication that *RANK* and *RANKL* may use in the diagnosis of the BMD phenotypes has emerged. Through multivariate analyses, we were able to detect a possible involvement of the combined *RANKL* (GG)–*RANK* (CC) genotype in defining the BMD level, but no contribution was observed for OPG variants. Recently, it has been shown that the coexistence of low concentration of RANKL and the C allele at the FokI polymorphic site of the VDR gene was strongly associated with pure disc herniation [20]. They concluded that a specific genetic association with the low bone turnover rate in patients affected by lumbar disc herniation could be one of the favouring factors for disc degeneration.

We assessed postmenopausal subjects diagnosed with low BMD or osteopenia from Saudi Arabia which has not previously been studied. The polymorphisms in the three genes (RANK, RANKL, and OPG) studied by us known to be involved in the maintenance of BMD. Previously, OPG gene polymorphisms were considered as a risk factor for postmenopausal osteoporosis [21, 22], whether it has an association with osteopenia among postmenopausal subjects has not been well studied. Previous reports have not shown any remarkable differences among the circulatory concentration of the RANKL, OPG, or RANKL/OPG ratio and their genotypes [23, 24]. Although bone resorption and osteoclasts function require balance activity of RANKL and OPG, however, the various factors involve for the regulation of *OPG*, *RANK*, and *RANKL* expression [25]. The SNPs in *RANKL* were found to have an association with a decreased level of BMD in European individuals [10]. SLE patient having low BMD had a high prevalence of the *OPG* 245 T>G which affirms the association of this SNP with low BMD [21, 22]. Possibly 245 T>G polymorphism in *OPG* may cause its dysfunction or inactivation which leads to having an adverse effect on BMD, although the OPG serum concentration remained normal [26]. However, no association of *OPG* 1181G>C, *OPG* 163A>G, and *RANK* A>G polymorphisms was found between BMD and vertebral fractures in premenopausal SLE patients [27], although these genetic variations were associated with lower BMD and a higher risk of osteoporosis in postmenopausal women [15, 28, 29]. In the current study, we only studied the association of these genes with postmenopausal osteopenia subjects. Polymorphism in *RANK* showed positive contributing factor for osteopenia while *RANKL* polymorphism may also contribute to bone mineral pathology. No association of *OPG* polymorphism was noted in osteopenia subjects. That may be either because of low sample size or individually, it may not contribute to BMD.

## 5. Conclusions

In summary, the polymorphism in *RANKL* and *RANK* may be associated with low BMD in Saudi postmenopausal women with osteopenia. *OPG* did not show association with osteopenia in the current subjects. Further studies that consider bone turnover markers or additional confounding factors are needed for testing in a larger sample size.

## Figures and Tables

**Table 1:** Characteristics of participants according to osteopenia status.

Parameters	Control	Osteopenia	*p* values
*N*	216	223	
Anthropometrics			
Age (years)	53.5 ± 6.0	55.8 ± 8.0	0.001
Age of menopause (years)	4.0 (2.0-7.0)	6.0 (3.0-10.0)	<0.001
BMI (kg/m^2^)	34.1 ± 5.4	33.5 ± 5.7	0.26
WHR	0.9 ± 0.1	0.9 ± 0.1	0.99
Systolic BP (mmHg)	124.8 ± 17.8	130.0 ± 18.3	0.004
Diastolic BP (mmHg)	75.3 ± 11.1	76.8 ± 12.2	0.19
Glucose (mmol/l)	7.8 ± 3.2	7.7 ± 3.2	0.68
Insulin (ng/ml)	0.47 (0.31-0.68)	0.55 (0.32-0.98)	0.44
Lipids			
Total cholesterol (mmol/l)	4.9 ± 0.9	5.0 ± 1.0	0.15
Triglycerides (mmol/l)	1.6 (1.2-2.3)	1.6 (1.2-2.3)	0.77
HDL-cholesterol (mmol/l)	1.1 ± 0.3	1.1 ± 0.3	0.72
Bone turnover markers			
*T*-score (AP spine)	-0.3 (-0.7-0.3)	-1.7 (-2.1--1.2)	<0.001
*T*-score (dual femur left)	0.7 (0.0-1.3)	-0.5 (-1.1-0.3)	<0.001
25(OH)D (nmol/l)	60.6 (35.4-82.7)	61.3 (38.8-89.2)	0.70
VDBP (*μ*g/ml)	11.0 (5.9-46.6)	14.9 (4.7-54.5)	0.76
PTH (pg/ml)	11.0 (6.6-19.1)	15.4 (9.6-27.5)	0.004
DKK1 (ng/ml)	3.1 (1.98-4.1)	3.1 (2.1-3.8)	0.88
OPG (ng/ml)	0.72 (0.50-0.95)	0.78 (0.58-1.07)	0.14
OPN (ng/ml)	2.3 (1.3-3.4)	2.5 (1.4-3.4)	0.78
RANKL (pg/ml)	34.3 (20.6-66.3)	24.0 (18.5-39.4)	0.001
SOST (ng/ml)	1.4 (0.61-2.7)	1.6 (0.8-2.3)	0.95
Osteocalcin (ng/ml)	8.8 (2.9-13.1)	8.7 (3.4-14.0)	0.71
ß-Crosslap (ng/ml)	0.1 (0.0-0.1)	0.1 (0.0-0.1)	0.11
Total pyridinoline (ng/ml)	15.1 (7.0-31.3)	15.5 (7.5-29.4)	0.90
NTx (nmol/l)	49.1 (37.0-64.7)	55.9 (40.9-84.7)	0.06
Interleukins			
IL6 (pg/ml)	7.5 (3.2-25.5)	12.1 (6.4-25.6)	0.013
IL1B (pg/ml)	1.6 (0.4-2.7)	2.0 (0.3-2.7)	0.39
IL4 (pg/ml)	7.7 (4.7-10.4)	6.5 (3.2-10.0)	0.15
Adipocytokines			
Leptin (ng/ml)	16.9 (8.1-33.2)	19.7 (8.4-37.0)	0.38
TNF-*α* (pg/ml)	1.6 (1.1-2.3)	1.9 (1.4-2.6)	0.032
Growth factors			
FGF23 (pg/ml)	62.6 ± 24.2	65.3 ± 23.2	0.30
TGF *β* (ng/ml)	41.2 (35.1-52.8)	38.4 (25.7-47.6)	0.06
IGF-1 (ng/ml)	17.8 (12.1-43.0)	12.8 (11.4-18.5)	0.29

**Table 2:** Association between osteopenia and *RANK* SNP.

SNP	Control *N* (%)	Osteopenia *N* (%)	Osteopenia	
			OR (95% confidence interval)	*p* value
rs1805034				
TT	80 (50.6)	15 (9.5)	1	
TC	96 (46.6)	22 (10.7)	1.22 (0.60–2.51)	0.58
CC	27 (39.7)	12 (17.6)	2.37 (1.00–5.69)	0.05
T	256 (63.1)	52 (53.1)	1	
C	150 (36.9)	46 (46.9)	1.51 (0.97–2.36)	0.07
rs35211496				
CC	166 (47.6)	41 (11.7)	1	
TC	29 (44.6)	7 (10.8)	0.98 (0.40–2.39)	0.96
TT	1 (33.3)	0 (0)	--	--
C	361 (92.1)	89 (92.7)	1	
T	30 (7.9)	7 (7.3)	0.92 (0.39–2.15)	0.84

Note: data are presented as frequencies *N* (%) and OR (95% CI). *p* value <0.05 is considered significant.

**Table 3:** Association between osteopenia and *RANKL* SNP.

SNP	Control *N* (%)	Osteopenia *N* (%)	Osteopenia	
			OR (95% confidence interval)	*p* value
rs2277438				
GG	10 (5.0)	70 (48.3)	20.56 (9.82–43.06)	<0.001
AG	49 (24.5)	27 (18.6)	1.62 (0.91–2.87)	0.09
AA	141 (70.2)	48 (33.1)	1	
G	69 (17.3)	167 (57.5)	4.54 (3.18–6.49)	<0.001
A	231 (82.7)	123 (42.4)	1	
rs9533156				
TT	92 (36.9)	79 (31.7)	1	
TC	82 (31.3)	97 (37.0)	1.38 (0.91–2.10)	0.14
CC	42 (38.5)	39 (35.8)	1.08 (0.64–1.84)	0.77
T	266 (61.6)	255 (59.3)	1	
C	166 (38.4)	175 (40.7)	1.10 (0.84–1.44)	0.50

Note: data are presented as frequencies *N* (%) and OR (95% CI). *p* value <0.05 is considered significant.

**Table 4:** Association between osteopenia and *OPG* SNP.

SNP	Control	Osteopenia	Osteopenia	
			OR (95% confidence interval)	*p* value
rs2073618				
GG	20 (43.5)	3 (6.5)	0.44 (0.12–1.59)	0.21
CG	93 (53.4)	17 (9.8)	0.54 (0.28–1.04)	0.07
CC	85 (42.3)	29 (14.4)	1	
G	133 (33.6)	23 (23.5)	0.61 (0.36–1.01)	0.06
C	263 (66.4)	75 (76.5)	1	
rs3102735				
TT	133 (46.5)	35 (12.2)	1	
TC	37 (50.0)	10 (13.5)	1.03 (0.47–2.27)	0.95
CC	1 (47.0)	0(0)	--	--
T	303 (88.6)	80 (88.9)	1	
C	39 (11.4)	10 (11.1)	0.97 (0.47–2.03)	0.94

Note: data are presented as frequencies *N* (%) and OR (95% CI). *p* value <0.05 is considered significant.

## Data Availability

Data will be made available upon reasonable request.
